# Efficient Single-Gene and Gene Family Editing in the Apicomplexan Parasite *Eimeria tenella* Using CRISPR-Cas9

**DOI:** 10.3389/fbioe.2020.00128

**Published:** 2020-02-25

**Authors:** Dandan Hu, Xinming Tang, Choukri Ben Mamoun, Chaoyue Wang, Si Wang, Xiaolong Gu, Chunhui Duan, Sixin Zhang, Jinxia Suo, Miner Deng, Yonglan Yu, Xun Suo, Xianyong Liu

**Affiliations:** ^1^Key Laboratory of Animal Epidemiology and Zoonosis, Ministry of Agriculture, National Animal Protozoa Laboratory, College of Veterinary Medicine, China Agricultural University, Beijing, China; ^2^Department of Internal Medicine and Microbial Pathogenesis, School of Medicine, Yale University, New Haven, CT, United States; ^3^Department of Clinical Veterinary Medicine, College of Veterinary Medicine, China Agricultural University, Beijing, China

**Keywords:** *Eimeria tenella*, CRISPR-Cas9, genetic engineering, apicomplexa, ApiAp2

## Abstract

*Eimeria* species are pathogenic protozoa with a wide range of hosts and the cause of poultry coccidiosis, which results in huge economic losses to the poultry industry. These parasites encode a genome of ∼8000 genes that control a highly coordinated life cycle of asexual replication and sexual differentiation, transmission, and virulence. However, the function and physiological importance of the large majority of these genes remain unknown mostly due to the lack of tools for systematic analysis of gene functions. Here, we report the first application of CRISPR-Cas9 gene editing technology in *Eimeria tenella* for analysis of gene function at a single gene level as well as for systematic functional analysis of an entire gene family. Using a transgenic line constitutively expressing Cas9, we demonstrated successful and efficient loss of function through non-homologous end joining as well as guided homologous recombination. Application of this approach to the study of the localization of EtGRA9 revealed that the gene encodes a secreted protein whose cellular distribution varied during the life cycle. Systematic disruption of the ApiAp2 transcription factor gene family using this approach revealed that 23 of the 33 factors expressed by this parasite are essential for development and survival in the host. Our data thus establish CRISPR-Cas9 as a powerful technology for gene editing in *Eimeria* and will set the stage for systematic functional analysis of its genome to understand its biology and pathogenesis, and will make it possible to identify and validate new targets for coccidiosis therapy.

## Introduction

*Eimeria tenella*, the causative agent of the intestinal disease coccidiosis, is an apicomplexan protozoan parasite of critical importance in veterinary medicine, and most notably in poultry. The parasite disrupts the intestinal epithelial cells of young poultry causing hemorrhagic cecal disease and even death (Chapman et al., 2013), which results in major economic losses worldwide (Blake and Tomley, 2014). The disease is thus considered a major risk to animal health of poultry. *E. tenella* undergoes a complex life cycle in the cecal epithelium of chicken following ingestion of sporulated oocysts. Three cycles of schizogony and a subsequent gametogony occur before unsporulated oocysts are shed with feces and undergo sporulation (Chapman et al., 2013).

Genome sequencing revealed that the life cycle of *E. tenella* is controlled by more than 8000 genes (Reid et al., 2014). While significant progress has been achieved over the past several years to understand the function of some of these genes in *E. tenella* development, differentiation, virulence, and susceptibility to therapy, the large majority of its genes remain inaccessible and their function unknown (Reid et al., 2014; Blake, 2015). Functional analysis in *Eimeria* genes has so far been limited to expression of recombinant proteins and localization of these proteins by indirect fluorescence microscopy. Although the first transfection of *Eimeria* was reported 20 years ago (Kelleher and Tomley, 1998), and several exogenous antigens have been successfully expressed in the parasite for the purpose of generating transgenic lines that could be used as vaccine strains (Pastor-Fernández et al., 2018; Tang et al., 2018a, b), no genetic editing tools that could be used in large-scale and systematic functional analysis have been developed heretofore.

Clustered regularly interspaced short palindromic repeats (CRISPR) is a system for DNA recognition used as a defense mechanism in bacteria and archaea (Rodolphe et al., 2007). In recent years, the type II CRISPR system from *Streptococcus* has been successfully used to introduce double-stranded breaks (DSBs) in the genomic DNA of several protozoan parasites including *Plasmodium* (Mehdi et al., 2014; Wagner et al., 2014), *Toxoplasma* (Shen et al., 2014; Sidik et al., 2014), *Leishmania* (Sollelis et al., 2015; Zhang and Matlashewski, 2015), *Trypanosoma* (Peng et al., 2015; Medeiros et al., 2017), and *Cryptosporidium* (Vinayak et al., 2015). In this system, the targeted genome cleavage is achieved by an RNA–Protein complex consisting of a customizable 20 nucleotide sequence that directs a Cas9 endonuclease to the genome location of interest through RNA–DNA hybridization (Jinek et al., 2012). DSBs introduced by Cas9 are then repaired by the cellular machinery through either non-homologous end-joining pathway (NHEJ), creating insertions or deletions (indels), or homology-directed repair (HDR) if an appropriate DNA template is provided (Haber, 2000). Due to the lack of a continuous *in vitro* culture system (Bussière et al., 2018) for *Eimeria* and a low transfection efficiency in this parasite (Clark et al., 2008; Liu et al., 2008), alternative strategies to co-transfection of two plasmids containing Cas9-gRNA cassettes and donor DNA or to the use of a single but large plasmid constructs are thus critical to a successful implementation of the CRISPR-based gene editing approach in this parasite. Interestingly, studies in mammalian cells and other parasites have demonstrated successful stable expression of the Cas9 protein (Koike-Yusa et al., 2014; Peng et al., 2015; Sidik et al., 2016) as a strategy to enable genome-wide gene loss-of-function screening and to circumvent the difficulty of gene delivery in species with low transfection efficiency.

To develop an efficient gene disruption tool for *Eimeria*, we first constructed a stable *E. tenella* parasite line that expresses the *S. pyogenes* Cas9(SpCas9) endonuclease. Using this line, we achieved efficient target gene disruption by introducing a single vector harboring the U6-gRNA and donor DNA. As a proof of principle, we implemented this approach to tag the putative *E. tenella* dense granule protein 9 (EtGRA9), and monitored its expression throughout the life-cycle of the parasite. To further assess the application of this strategy for large-scale and systematic analysis of the *E. tenella* genome, we applied the CRISPR-Cas9-based technology to disrupt each of the 33 members of the AP2 transcription factors family. To the best of our knowledge, this is the first successful targeted gene disruption in *Eimeria* species. This data will set the stage for large-scale and systematic functional analysis of the *E. tenella* genome to help unravel the physiological function of the ∼8000 genes encoded by *E. tenella* in parasite’s development, differentiation, virulence, and susceptibility to antimicrobials.

## Results

### Generation of a Stable Cas9-Expressing Transgenic *E. tenella* Line

**FIGURE 1:**
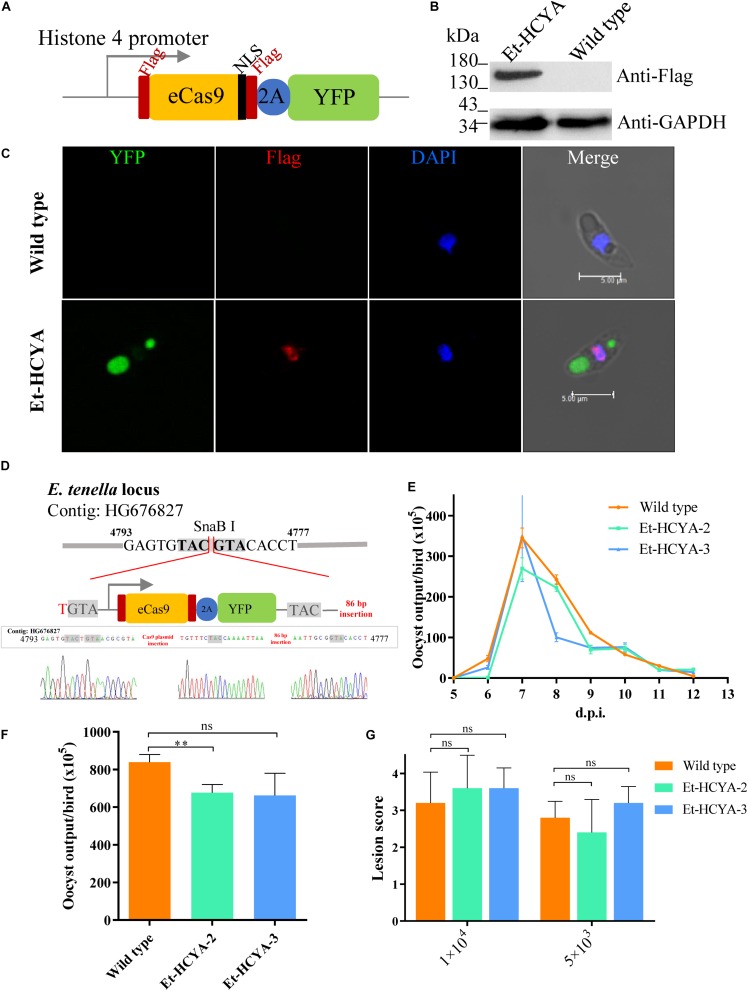
Construction and identification of Cas9-expressing Et-HCYA lines. **(A)** Schematic illustration of plasmid expressing eCas9 and YFP separated by a “self-cleavage” 2A peptide. Stably expression of Cas9 protein in Et-HCYA was identified by Western blot **(B)** and immunofluorescent assay **(C)** using mouse anti-Flag mAb. **(D)** Schematics of the insertion site of Cas9 plasmid. The insertion site was determined by genome-walking and identified by PCR and sequencing. Oocyst output curves **(E)** and total oocyst outputs **(F)** of eCas9-expressing lines. Chicken (*n* = 4) were infected with 1000 oocysts, and oocyst outputs were detected daily in triplicates. **(G)** Intestine lesion scores after chickens were infected with eCas9-expressing clones. Chickens (*n* = 5) were infected with 5000/10,000 oocyst/bird, and the lesion scores were scored 7 d.p.i. ns, not significant; ^∗∗^*p* < 0.01.

**FIGURE 2:**
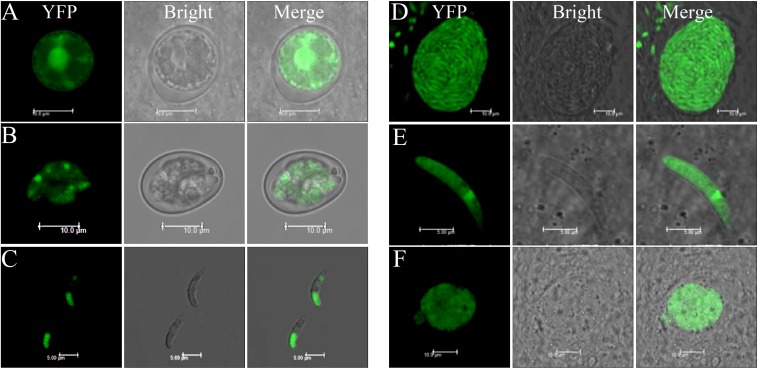
Cas9 protein expression in all stages of EtHCYA. Chicken were infected with 10,000 Et-HCYA clone #3, then the cecal smears were prepared at 96, 120, and 156 h.p.i. for the detection of merozoites, gametocytes, and unsporulated oocysts, respectively. Oocysts were collected and purified from feces. Sporozoites were purified from sporulated oocysts. **(A)** Unsporulated oocyst; **(B)** sporulated oocyst; **(C)** sporozoites; **(D)** schizont; **(E)** merozoite; and **(F)** gametocyte.

Although the humanized SpCas9 sequence has been used in other apicomplexan parasites including *T. gondii*, our attempts to use it in *E. tenella* was not successful, likely due to weak expression of the transgene (data not shown). Therefore, we optimized the sequence of SpCas9 using the *E. tenella* codon usage preference. The 4203 bp optimized Cas9 (eCas9) nucleotide sequence was synthesized and cloned downstream of the Histone 4 gene promoter to generate the eCas9-NLS-2A-YFP transfection vector (Figure 1A). In this construct, eCas9 harbors a C-terminal nuclear targeting sequence and two Flag tags and is fused to the reporter molecule YFP with the two sequences separated by the “self-cleavage” 2A peptide. Immunoblot analysis using a monoclonal anti-Flag antibody showed successful expression of eCas9 in *E. tenella* as a ∼156 kDa protein (Figure 1B). To further localize eCas9 in transgenic parasites, *E. tenella* sporozoites were stained with the anti-Flag monoclonal antibody, and examined by confocal microscopy. As shown in Figure 1C, the fluorescence signal for eCas9 expression co-localized with the nuclear stain DAPI, whereas the YFP signal was confined to the large and small refractile bodies of the parasite. As expected, the expression of eCas9-YFP was constitutive throughout the life cycle of the parasite (Figure 2).

To generate a genetically stable eCas9-expressing *E. tenella* line, single sporocysts were isolated by FACS and inoculated into chicken. Two clones were selected following infection of 35 chicken with single sporocysts. Analysis of these two transgenic clones showed little to no differences in total oocyst output compared to the isogenic wild type strain and overall similar oocyst output curve, and intestine lesion score as the isogenic parental line (Figures 1E–G).

To identify the insertion site of the Cas9 cassette in the *E. tenella* genome, total genomic DNA from the Et-HCYA transgenic line (clone #3) was extracted and used for genome walking using specific primers (Supplementary Table S1). Sequence analysis identified the site of insertion to be in an intergenic region between nucleotides 4777 and 4793 of the *E. tenella* genomic contig HG676827 with one base pair insertion in the N-terminal and 86 bp insertion on the C-terminal region of the inserted plasmid, respectively (Figure 1D).

### Site-Specific Cas9-Mediated Mutagenesis in *E. tenella*

**FIGURE 3:**
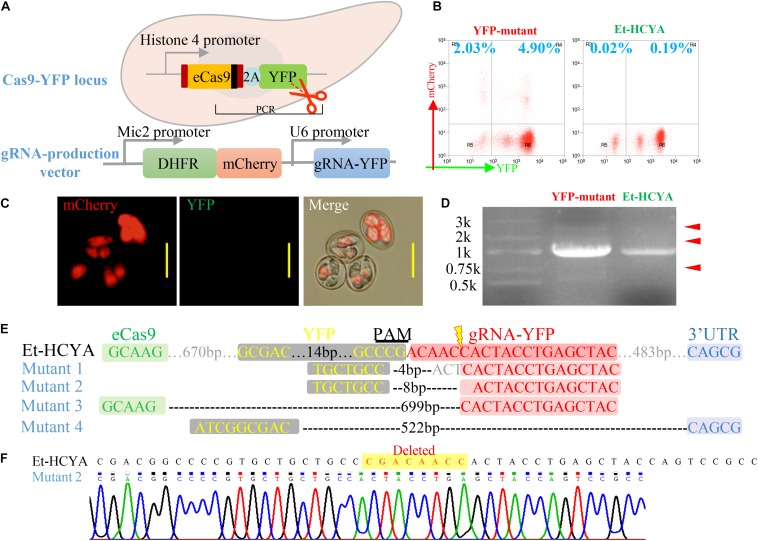
CRISPR/Cas9-mediated YFP mutagenesis in transgenic EtHCYA. **(A)** Schematics showing the mutagenesis strategy by transfecting a vector encoding a gRNA targeting endogenous *yfp* locus in Et-HCYA. **(B)** The mutation rate of the transfected oocysts was analyzed by flow cytometry. Left: Et-HCYA transfected with gRNA-producing vector; Right: Et-HCYA control. **(C)** Fluorescence observation of mutant oocysts under microscope. Bar = 20 μm. **(D)** Cleaved *yfp* loci in the transfected population were identified by PCRs, and all the mutated amplicons (red arrowhead) were cloned and sequenced. **(E)** All detected mutations in the *yfp* loci induced by Cas9 cleavage. **(F)** Chromatogram of mutant 2.

To examine the efficiency of double strand DNA cleavage activity of eCas9 in eCas9-expressing *Eimeria*, the Et-HCYA line was transfected with gRNA-expressing plasmid targeting the endogenous *yfp* locus from eCas9-NLS-2A-YFP plasmid (Figure 3A). The vector harbors the DHFR-TS selectable marker (*T. gondii* bifunctional dihydrofolate reductase-thymidylate synthase mutant which confers resistance to pyrimethamine), the mCherry reporter under the Mic2 promoter, and the YFP guide RNA under the control of the U6 promoter. Following transfection, ∼7% of oocysts analyzed by flow cytometry were found to express mCherry. Of these, ∼2% were negative for YFP fluorescence (Figures 3B,C), suggesting a cleavage efficiency of ∼29%. Analysis of the genomic DNA of the transfectants by PCR, to detect the targeted sites repaired by NHEJ, identified multiple bands of different lengths. As expected, the control Et-HCYA line showed a single band (Figure 3D). Subsequent cloning and sequencing of all the bands identified in the transfectants revealed four distinct deletions of 4, 8, 522, and 699 bp (Figures 3E,F), all occurring near the cleavage target site and the protospacer adjacent motif (PAM, Figure 3E).

### Cas9-Mediated Gene Disruption in *E. tenella*

**FIGURE 4:**
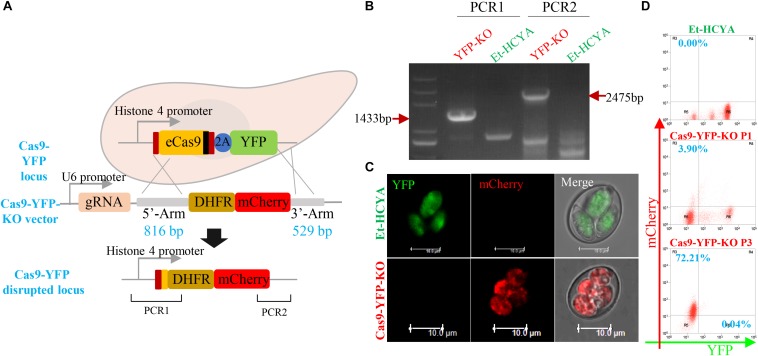
CRISPR/Cas9-directed YFP gene disruption in transgenic EtHCYA. **(A)** Schematics showing CRISPR/Cas9-mediated disruption of endogenous *cas9-yfp* locus by transfecting Cas9-YFP-KO vector. This vector contains a gRNA targeting *yfp* driven by EtU6 promoter, and DHFR:mCherry coding sequence (without promoter) flanked by 5′ and 3′ homologous regions. Endogenous *cas9-yfp* locus disruption was determined by PCR **(B)** and fluorescent microscopy **(C)**. The specific bands in Cas9-YFP-KO parasites were marked with red arrows. **(D)** Cas9 editing efficiency detected by flow cytometry. P1: first generation of progeny oocysts post-transfection; P3: third generation post-transfection.

To evaluate whether eCas9 can mediate double-stranded gene break and homologous recombination in *E. tenella*, the eCas9-expressing sporozoites were transfected with a plasmid containing the YFP gRNA as well as 5′ and 3′ fragments of homology to sequences in the eCas9-Flag-2A-YFP vector (Figure 4A). Because the targeting construct was designed so that the DHFR-mCherry cassette lacks a promoter region, only successful gene replacement by homologous recombination would result in expression of the selectable marker under the control of the Histone 4 promoter that otherwise drives the expression of the eCas9 cassette in the Et-HCYA line (Figure 4A). Flow cytometry analysis of the resulting transfectants showed a 67% loss of the YFP signal in oocysts compared to the parental strain and identified ∼4% of oocysts that express mCherry fluorescence only (Figures 4C,D). These findings suggest that the eCas9-mediated cleavage occurs with a high efficiency in *E. tenella*, but that the recombination rate is low. Cell sorting of the mCherry^+^ YFP^–^ sporocysts and subsequent passaging produced a homogenous *E. tenella* population of mCherry^+^ YFP^–^ transfectants (Cas9-YFP-KO line) by the third generation as demonstrated by flow cytometry, PCR, and sequencing (Figure 4B).

Thirty-two potential off-targets were found in the Et-HCYA genome with maximum mismatch number of five nucleotides using the Cas-OFFinder software (Supplementary Table S2). Sequencing of the genome of Et-HCYA and Cas9-YFP-KO line to detect variants identified 138 single nucleotide variations (SNVs) in the Cas9-YFP-KO line. However, none of these mutations were found within the 2 kb region surrounding the predicted off-target sites. Altogether, these data suggest that these SNVs are not the result of Cas9 cleavage but could have emerged from random mutations during the three consecutive passages in chicken.

### CRISPR/Cas9-Mediated Gene Tagging of *E. tenella* EtGRA9 Secretory Protein

**FIGURE 5:**
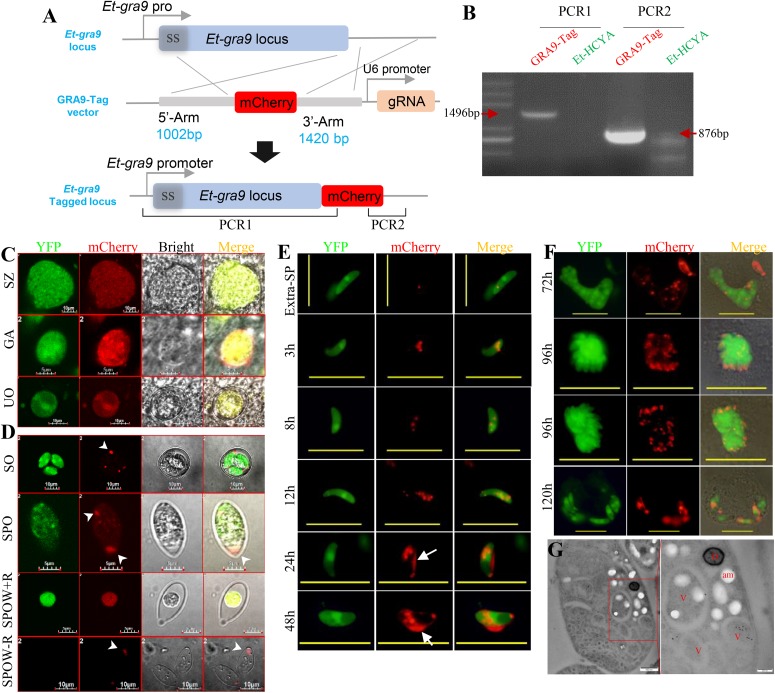
C-terminal tagging of *Etgra9* mediated by CRISPR/Cas9 and the observation of expression pattern of tagged *Etgra9* in parasites. **(A)** Schematics showing CRISPR/Cas9-mediated targeting of *Etgra9* gene. Et-HCYA were transfected with GRA9-Tag vector, which contains a U6-gRNA cassette and a homologous recombination cassette with mCherry fused to the C-terminal of *Etgra9* gene. **(B)** Oocysts with yellow and mCherry fluorescence were sorted by FACS, then genomic DNA was extracted for PCR identification. **(C)** Localization of mCherry-tagged EtGRA9 in different developmental stages *in vivo*. The cecal smears were prepared at 96, 120, and 156 h.p.i. for the detection of merozoites, gametocytes, and unsporulated oocysts, respectively. UO, unsporulated oocyst; SZ, schizont; and GA, gametocyte. **(D)** Localization of mCherry-tagged EtGRA9 during excystation. Sporocyst (SPO) were released from sporulated oocyst (SO) by glass beads grinding, and sporozoites (Extra-SP) were excysted after bile-trypsin digestion, resulting empty sporocyst wall with (SPOW + R)/without (SPOW–R) residue body. Sporozoites were observed at different time points after being inoculated onto MDBK cells **(E,F)**. White arrowheads show EtGRA9 in Stieda bodies and dense granule; white arrows show EtGRA9 secreted into the parasitophorous vacuole. The localization of EtGRA9 was also observed by immune-electron microscopy **(G)**. The ultrathin sections of purified second generation merozoites were labeled with anti-mCherry antibody and followed by immunogold labeling. D, dense granule; V, vesicle; am, amylopectin. Bars in panels **C**, **D**, and **G** are indicated in graph, and bars are 20 μm in panels **E** and **F**.

Studies in *T. gondii* have shown that the dense granule protein TgGRA9 associates with the vacuolar network membranes (Adjogble et al., 2004). To evaluate the expression pattern of the putative *Etgra9* gene in *E. tenella* development, we applied the Cas9-mediated approach. The EtGRA9 protein (ToxoDB ID: ETH_00028350) shares 25% similarity with the *T. gondii* GRA9, contains a 23 aa signal peptide and a putative *N*-palmitoyl cysteine. To monitor the expression profile of EtGRA9 during *E. tenella* life cycle, the C-terminal region of the protein was tagged with mCherry by Cas9-mediated homology recombination in the Et-HCYA line (Figure 5A). Cell sorting of sporocysts after subsequent passages produced a GRA9:mCherry line, which was further validated for correct expression of the gene under the endogenous promoter by PCR and sequencing (Figure 5B). Fluorescence microscopy showed EtGRA9 to be expressed in all *E. tenella* stages, and to be uniformly distributed in unsporulated oocysts, merozoites, and gametocytes *in vivo* (Figure 5C). In sporocysts, however, EtGRA9 was primarily targeted to the Stieda bodies and dotted distributed (Figures 5D,E). Following sporozoite excystation, two localization patterns were found. Sporocysts containing the residue body showed localization of EtGRA9 to this organelle, whereas those lacking the residue body showed localization of EtGRA9 to the Stieda body (Figure 5D). GRA9:mCherry sporozoites were also cultured in Madin-Darby Bovine Kidney (MDBK) cells to monitor protein localization following sporozoite invasion. EtGRA9 was observed as a single dot in the extra cellular sporozoites, while the protein was dotted distributed in a few more number after 3 h post-invasion. By 24 h post-invasion, the protein could be found in a greater number of dots and the parasitophorous vacuole (PV). EtGRA9 was subsequently distributed in the immature multi-nucleated schizont, and then localized to each merozoite (Figure 5F). Following merozoite released and invasion of new cells, EtGRA9 was then redirected to the PV. Immuno-electron microscopy was used to identify the details of these EtGRA9 containing dots in the merozoites. To our surprise, the EtGRA9 labeling signal was not found in the dense granule but in the vesicles (Figure 5G). These results suggest that the EtGRA9 is a secreted protein that may help *E. tenella* sporozoites release from sporocysts, and may play an important role during invasion by sporozoites and merozoites. Interestingly, whereas tagging of the endogenous *Etgra9* gene could be easily achieved, no knock-out lines could be isolated using the eCas9-mediated gene disruption, suggesting that the gene is essential for parasite’s viability.

### Cas9-Mediated Gene Disruption for Functional Analysis of 33 *E. tenella* AP2 Family Genes

**FIGURE 6:**
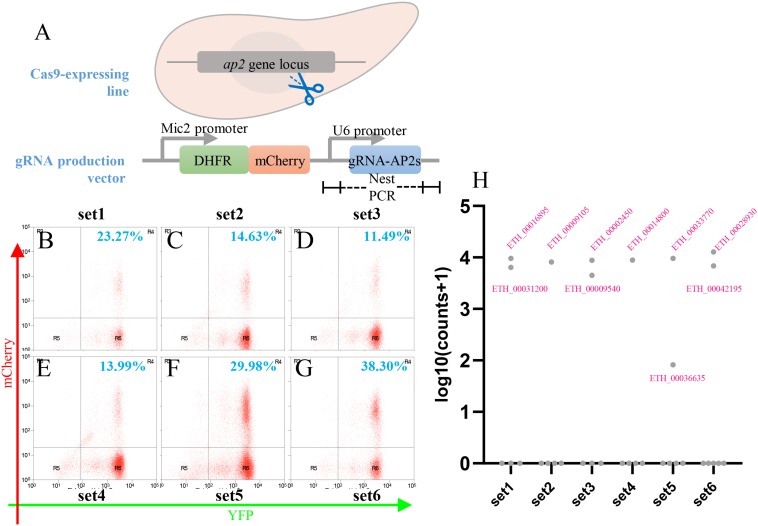
Cas9-mediated *Etap2* family gene disruption. **(A)** Schematics showing the *Etap2* family genes disrupted by transfection with gRNA-producing vectors. **(B–G)** Thirty-three *Etap2* genes were screened by six sets of transfections, and the efficiency of each transfection was informed by flow cytometry analysis. **(H)** Log10 abundances of gRNAs remained in the positive oocysts determined by pooled sequencing of nest-PCR amplicons. Target gene IDs were indicated for their gRNAs presented in the sequencing reads.

Studies in other protozoan parasites showed an important role for AP2 transcription factors in virulence, sexual commitment, and development (Painter et al., 2011; Radke et al., 2013; Kafsack et al., 2014; Sinha et al., 2014). Our analysis of the *Eimeria* genome identified a family of 33 AP2 genes encoding proteins with the AP2 domain. To establish the systematic analysis of this gene family in *E. tenella*, we designed guide RNAs targeting each of the 33 AP2 genes (see Supplementary Table S3). These were then cloned under the regulatory control of the U6 promoter in the eCas9 targeting vector and the resulting constructs used to conduct six independent transfections of the eCas9-expressing *E. tenella* line Et-HCYA (Figure 6A). Following transfection, viable mCherry^+^ parasites were selected by FACS and analyzed by PCR, high-throughput sequencing, and read mapping to identify the gRNAs present in viable transfectants (Figures 6B–G). Interestingly, of the 33 AP2 genes targeted for disruption, only 10 could be successfully targeted as determined by the presence of the gRNAs (Figure 6H). Of these, nine gRNAs were readily detectable after eCas9 cleavage. These gRNAs target the following AP2 genes: ETH_00016895, ETH_00031980, ETH_00009105, ETH_00002450, ETH_00009540, ETH_00014800, ETH_00033770, ETH_00028930, and ETH_00042195. The 10th gRNA targeted ETH00036635 and was associated with a low read count, suggesting that disruption of this gene results in a defect in parasite’s growth (Figure 6H). Together, these data suggest that of the 33 AP2 transcription factors of *E. tenella*, 23 are essential to the parasites, and 10 are dispensable. These findings will set the stage for future detailed phenotypic analyses of viable mutants as well as genetic analyses by conditional disruption of essential genes to assess which stages of *E. tenella* life cycle they control.

## Discussion

The lack of genetic tools for functional analysis of *Eimeria* parasites is a major obstacle to the advancement of the biology of these important protozoa and to efforts aimed to control coccidiosis. In this study, we report the first successful application of CRISPR-Cas9-mediated gene editing in *Eimeria* and provide evidence for its use for loss-of-function analysis of the AP2 transcription factor family. We constructed a stable Cas9-expressing *E. tenella* line that undergoes a life cycle that is indistinguishable from that of the wild-type parasite. Using this transgenic line, we found that the cleavage activity of Cas9 occurs with high efficiency. We also used this line to demonstrate successful deletion of the *yfp* gene inserted into the chromosome as well as successful tagging of the EtGRA9 secreted protein. Moreover, we provide evidence that the technology can be applied to target the 33 ApiAp2 transcription factors encoded by *E. tenella* to determine which ones are essential for development and survival of the parasite.

Although our data represent a major advance in *E. tenella* genetic manipulation, further optimizations are needed to streamline the use of this technology for gene editing in this parasite. First, although some progress has been made in the *in vitro* culture of *Eimeria* (Shi et al., 2008; Bussière et al., 2018), the majority of cultured parasites do not complete their life-cycle. Thus, cloning of single parasites requires propagation of transgenic parasites in animals. This strategy, however, is not very efficient as only ∼10% of the clones could be isolated by *in vivo* propagation. Second, unlike trypanosomatids that lack an NHEJ pathway (Burton et al., 2007; Glover et al., 2010), *Eimeria* species repair DSB DNAs mainly by NHEJ pathway because of the conserved KU70-/KU80-dependent mechanism. This problem, however, has recently been solved in *T. gondii* by deleting the NHEJ repair pathway through disruption of the KU80 coding gene (Fox et al., 2009) or by using Cas9-mediated DSB followed by cloning single tachyzoites (Shen et al., 2014; Sidik et al., 2014). Finally, the low transfection efficiency in *Eimeria* (∼0.2%) (Clark et al., 2008; Liu et al., 2008) significantly limits the use of strategies that employ co-transfection of multiple plasmids, such as Cas9-gRNA plasmid and donor plasmid.

Interestingly, unlike our results in *E. tenella*, stable expression of Cas9 protein in *T. cruzi* (Peng et al., 2015) and *T. gondii* (Sidik et al., 2016) reduced the fitness of these parasites. The Cas9 protein altered the growth kinetics of *T. cruzi*, and this alteration could be rescued by deleting the Cas9 gene integrated in the *T. cruzi* genome (Peng et al., 2015). Constitutively expressed Cas9 protein is also lethal to *T. gondii*, and a “decoy” gRNA was necessary to reduce partially the toxicity of Cas9-expression in *T. gondii* (Sidik et al., 2016). According to Markus et al. (2019), viral 2A peptides can greatly improve the stability of Cas9 expressed in *T. gondii* tachyzoites. Thus, in our study, we fused a 2A peptide to the C-terminal end of Cas9, which made it possible to achieve a stable and constitutive Cas9-expression in *E. tenella* (in all life stages) without an impact on parasite development.

The CRISPR-Cas9 strategy developed in this study has many advantages. For example, it can reduce the time needed to construct Cas9 plasmids, and eliminates the need for co-transfection of a Cas9 plasmid and a donor vector, which may greatly reduce the editing efficiency. The strategy also enables possibilities in multi-gene family disruption by transfecting with gRNAs targeting conserved regions (Peng et al., 2015) or by multiple gRNA delivery (Sidik et al., 2016). Our analysis of the transgenic line identified limitations in the *Eimeria* system that need to be addressed in order to create a strategy amenable to large-scale functional analysis. *Eimeria* species repair their genome DSBs mainly by NHEJ, and that the ratio of random insertions in this parasite is higher than recombination when transfecting with linearized plasmid DNA. Another challenge is the lack of a reliable continuous *in vitro* culture system, which makes it difficult to clone individual parasites. Therefore, in order to avoid random insertions, we employed targeting constructs that harbor the DHFR-mCherry cassette without a promoter. Thus, only insertions resulting from homologous recombination result in expression of the maker–reporter fusion. However, this strategy might have limited application for genes whose expression is weak. Improvement of the *in vitro* culture system or disruption of the activity of the NHEJ pathway in *E. tenella* may help overcome this challenge.

Systematic functional analysis of *Eimeria* parasites could also benefit from strategies that employ a gene targeting strategy using PCR products rather than cloning. So far attempts to knock out the endogenous YFP in the Et-HCYA line by using DHFR:mCherry PCR products with 39 bp homology recombinant regions have not been successful. Further optimization of this approach is desirable and could pave the way for knockout of genes at a large scale. Recently, the CRISPR-Cas9 gene editing system in *Trypanosome* was optimized by transfecting parasites with ribonucleoprotein complexes consisting of the *S. aureus* Cas9 and gRNAs (Medeiros et al., 2017). Using this transfection strategy, a very high (near 100%) editing efficiency in *T. cruzi* and other kinetoplastids was achieved (Medeiros et al., 2017). Application of this strategy in *Eimeria* is also warranted.

In summary, we successfully adapted the CRISPR-Cas9 gene editing system for the genetic manipulation of *E. tenella* and created a transgenic line that constitutively express Cas9 without affecting the life cycle or fitness of the parasite. Using this line, we were able to manipulate both individual genes (*yfp* and *Etgra9*) as well as multiple genes (gene family of transcription factors in this study). It is anticipated that this toolbox will usher a new era in the functional genomic of *Eimeria* species, advance the biology of the parasite and help develop more effective strategies to control coccidiosis.

## Materials and Methods

### Ethics Statement

Use of animals in this study was approved by the Beijing Administration Committee of Laboratory Animals and performed in accordance with the China Agricultural University Institutional Animal Care and Use Committee guidelines [approval number: AW05(7)069102-2].

### Animals and Parasites

One- to six-week-old AA broilers used for *E. tenella* passages and transfection experiments were purchased from Beijing Arbor Acres Poultry Breeding (Beijing, China). Ten-day-old SPF chickens used in pathogenicity studies were purchased from Merial Animal Health Co., Ltd. (Beijing, China). All birds were fed with a coccidia-free diet and water *ad libitum*. The wild-type *E. tenella* Beijing strain used was maintained in the lab. The procedures for collection, purification, and sporulation of the parasite were carried out as previously described (Eckert, 1995).

### MDBK Cell Cultural and Parasite *in vitro* Infection

Madin-Darby Bovine Kidney cells were cultured as previously reported (Tierney and Mulcahy, 2003). *In vitro E. tenella* sporozoites infections were performed as described by Bussière et al. (2018). To remove the extra-cellular parasites, three times of wash with PBS were performed before sampling.

### Codon Optimization of SpCas9 Using *E. tenella* Optimal Codon Usage

For optimal expression of the SpCas9 protein in *E. tenella*, the gene was synthesized using *E. tenella* optimal codon usage, which was downloaded from Codon Usage Database^1^. Codon optimization was achieved using the online software OPTIMIZER^2^. The optimized Cas9 sequence could be found in Supplementary Data S1.

### Plasmid Construction

To construct the eCas9/YFP plasmid, the *eCas9* gene and the YFP gene encoding enhanced yellow fluorescent protein were expressed in a single cassette under the regulatory control of the Histone 4 promoter. The sequence was designed to also include a self-cleavage 2A peptide sequence between eCas9 and YFP.

To determine the cleavage activity of Cas9 in Et-HCYA line, a gRNA-producing plasmid (pgRNA) targeting YFP locus was constructed. In this plasmid, the mCherry sequence was fused to the pyrimethamine resistant gene TgDHFR-ts-m2m3 (DHFR) positive selectable marker and expressed under the control of the EtMic2 promoter. The plasmid also harbors the U6-gRNA cassette. The small guide RNA was designed using EuPaGDT^3^. The YFP-KO vector consisted of two cassettes, the gRNA was regulated by EtU6 promoter, and the DHFR positive selectable marker and mCherry. These sequences were flanked by 5′ (816 bp) and 3′ (529 bp) homology sequences. For the GRA9-tag vector, the coding region of mCherry was flanked by 5′ (gene sequence of *Etgra9*, 1002 bp) and 3′ (3′downstream of *Etgra9*, 1420 bp) homology sequences, followed by a gRNA cassette derived by EtU6.

For the genome-wide *Etap2* gene family disruption screen, *E. tenella ap2* genes were identified by searching against ToxoDB v42 using PF00847 and PF14733 with 1.0E−05 as threshold. Thirty-three *ap2* vectors were constructed based on pgRNA with substitution of gRNAs.

All PCR amplifications were performed using Q5^®^ High-Fidelity DNA Polymerase (New England Biolabs, Ipswich, MA, United States) and primers were listed in Supplementary Table S1. Elements in each plasmid were linked by seamless assembly strategy (ClonExpress MultiS One Step Cloning Kit, Vazyme Biotech Co., Ltd., Nanjing, China) based on pEASY-Blunt-Simple cloning vector (TransGen Biotech, Beijing, China) backbone with double SnaBI sites (for linearization) on the edge of inserts.

### Transfection and Establishment of Cas9-Expressing *E. tenella* Line

*Eimeria tenella* sporozoites were harvested and purified from freshly purified oocysts by Percoll centrifugation and bile-trypsin digestion (Dulski and Turner, 1988). The *Sna*BI-mediated transfection procedures were performed as previously reported (Qin et al., 2014). For *Et-ap2* gene family disruptions, five to six gRNA coffering plasmids were mixed as a group (six group in total), and 20 μg of each linearized plasmid was used for one transfection with ∼1 × 10^7^ sporozoites. These sporozoites were subsequently inoculated to six 2-week-old chicken through cloaca. Pyrimethamine (150 mg/L) was added to the drinking water with solubilize formula (kindly provided by Dr. Tuanyuan Shi from Zhejiang Academy of Agricultural Science, China).

For enrichment of positive transgenic parasites, sporocysts of the first-generation oocysts after transfection were extracted and purified as introduced previously (Dulski and Turner, 1988), and then sorted by flow cytometry. Briefly, for sporocyst isolation, purified sporulated oocyst were broken by vortexing with equal volume of 1 mm glass beads for 30 s (3000 rpm), then the broken oocyst pellets were resuspended with 50% percoll, remove the oocyst wall fragments in the up-layer after centrifuge for 1 min at 10,000 × *g*, then the sporocyst pellets were washed with PBS for three times. Single sporocyst was sorted and isolated to generate genetically stable Cas9-expressing *E. tenella* lines (Et-HCYA). To locate the insertion site of eCas9 plasmid in Et-HCYA #3 clone, genome walking experiments were performed according to the manufacture’s instruction (Takara Biomedical Technology, Beijing, China).

### Indirect Immunofluorescence Assay and Immunoblotting

Immunofluorescence assay (IFA) and immunoblotting were performed to confirm the expression of eCas9 in *E. tenella* as previously described (Tang et al., 2018a). Mouse anti-Flag monoclonal antibody (1:200) and Cy3-conjugated goat anti-mouse IgG (1:200) were used for the IFA and western blot analysis. Extracellular sporozoites were fixed with 4% formaldehyde for 10 min, and permeabilized with 0.25% Triton X-100 in PBS for 8 min and stained with anti-Flag antibody, and the nuclei were stained with DAPI.

Total oocyst proteins of Et-HCYA and wild strain were extracted for immunoblotting. Anti-Flag mAb (1:1000) and horseradish peroxidase (HRP)-conjugated goat anti-mouse IgG (1:2000) were used to detect Flag-tagged eCas9 protein. Anti-GAPDH mouse mAb (1:1000) was used as a control.

### Immuno-Electron Microscopy

Briefly, samples were fixed in 2% PFA and 0.2% GA overnight. After rinsing with phosphate buffer (PB) (0.1 M, pH 7.4), samples were dehydrated through a graded ethanol series (30, 50, 70, 80, 90, and 100%, 10 min each). Samples were infiltrated in a graded mixtures (3:1, 1:1, 1:3) of ethanol and LR White resin (Ted Pella) then changed two to three times pure resin. Finally, samples were embedded in pure resin and polymerized for 48 h at 50°C. The ultrathin sections (70 nm thick) were sectioned with microtome (Leica EM UC6). After rinsing with 0.1 M PB, sections were blocked in 5% goat serum in 1% BSA buffer (with 0.15% Glycine in PB) for 30 min, then incubated with 1:10 diluted Rabbit Anti-mCherry antibody (ab167453, Abcam, Cambridge, United Kingdom) for 2 h. After rinsing with 0.1 M PB six times again, sections were followed by immunogold labeled with 10-nm protein A-gold (1: 50; Cell Microscopy Center, University Medical Center Utrecht, Utrecht, Netherlands) for 1 h. Following rinses with PB, sections were stained with 2% uranyl acetate for 5 min, and imaged by a transmission electron microscope (FEI Tecnai Spirit 120kV).

### Oocyst Output Curves and Virulence Determination

The effect of eCas9 expression on the life cycle of *E. tenella* was determined by comparing the oocyst output and intestine damages between the Et-HCYA clones and the parental wild-type parent strain. One-week-old AA broilers (*n* = 4) were infected with 1000 fresh oocysts/bird/strain, oocyst outputs were measured daily during 5–12 days post infection (d.p.i.). Total oocyst outputs were also calculated by using a modified McMaster chamber (Haug et al., 2006). Ten-day-old SPF chicken were inoculated with 5000/10,000 fresh oocysts/bird (*n* = 5) and were then sacrificed at 7 d.p.i. for lesion scoring of the ceca (Johnson and Reid, 1970).

### High-Throughput gRNA Detection in EtAp2 Family Screen

2–3 × 10^5^ sporocysts of each group were sorted by FACS and mixed before infecting chicken for second generation merozoites. Genomic DNA was extracted from these merozoites, and the integrated gRNA sequences were amplified by nested PCR. Subsequently, the amplicons were used for illumina library construction using NEBNext^®^ Multiplex Small RNA Library Prep Set for Illumina^®^ kit (New England Biolabs, Ipswich, MA, United States) following the manufacturer’s instruction. The library was then used for Miseq using PE250 platform. The output reads were filtered and mapped to the gRNA library, and then the mapped reads were counted for each gRNA.

### Whole Genome Sequencing and Off-Target Evaluation

Second generation merozoites of Et-HCYA clone #3 and Cas9-YFP-KO line were isolated for gDNA extraction. Genome DNA were sheared into ∼300 bp fragments and were then processed and sequenced using illumina Novaseq 6000 platform according to the manufacturer’s protocol. Paired-end reads were filtered and then mapped to *E. tenella* reference genome (an unpublished new version) by bwa-mem, and the SAM files were sorted and converted to BAM files, and PCR duplications were also marked by GATK v4.0.11. The small nucleotide variations (SNVs) were called by freebayes v1.02 with parameters of “-C 5 –min-mapping-quality 30 –min-base-quality 20 –min-coverage 10” and samtools mpileup pipeline with default settings. The concordance SNVs called from the two methods were kept and used for filtration with “QUAL < 60.0, QD < 20.0, FS > 13.0, MQ < 30.0, MQRankSum < −1.65, ReadPosRankSum < −1.65.” In house python script was used to identify different SNVs between Et-HCYA and Cas9-YFP-KO lines. Potential off-target sites were predicted by Cas-OFFinder (Bae et al., 2014) with maximum five nucleotides mismatch, and the 2000 bp region around the potential off-target sites were checked for its mutation manually.

### Statistical Analysis

Unpaired multiple *t*-tests were used for the analysis in total oocyst output and lesion scores. All bar plots depict the mean with standard deviations shown as error bars.

## Data Availability Statement

The datasets generated for this study can be found in the NCBI via SRA accession: PRJNA587588: https://www.ncbi.nlm.nih.gov/sra/PRJNA587588.

## Ethics Statement

The animal study was reviewed and approved by the China Agricultural University Institutional Animal Care and Use Committee.

## Author Contributions

XL, XS, and CM conceived and designed the study. DH performed the experiments, analyzed the data, and drafted the manuscript. XT, CW, SW, XG, and CD helped in plasmid construction, data analysis, and manuscript writing. XT, SZ, JS, YY, and MD helped in animal experiments and oocyst collection. All authors read and approved the final manuscript.

## Conflict of Interest

The authors declare that the research was conducted in the absence of any commercial or financial relationships that could be construed as a potential conflict of interest.

## Footnotes

1 http://www.kazusa.or.jp/codon/cgi-bin/showcodon.cgi?species=5802

2 http://genomes.urv.es/OPTIMIZER/

3 http://grna.ctegd.uga.edu/

## Supplementary Material

TABLE S1 Primers used for this study (primers for plasmid construction are not listed).

TABLE S2 Potential off-target sites of Cas9-YFP-KO.

TABLE S3 Detailed read counts and other information for guide RNAs involved in this study. gRNAs were designed in EuPaGDT (http://grna.ctegd.uga.edu), and the top ranked gRNAs for each gene were selected for the transfection studies.

DATA S1 *E. tenella*-codon usage optimized nucleotide sequence of SpCas9.
