# Supplementary material for: Efficient Single-Gene and Gene Family Editing in the Apicomplexan Parasite Eimeria tenella Using CRISPR-Cas9
Source: Front Bioeng Biotechnol. 2020 Feb 25;8:128. doi: 10.3389/fbioe.2020.00128 (PMC7052334; doi:10.3389/fbioe.2020.00128)
Supplement: DATA S1 — E. tenella-codon usage optimized nucleotide sequence of SpCas9. [file Table_4.DOCX]

ATGGACAAGAAGTACAGCATTGGCCTGGACATTGGCACAAACAGCGTGGGCTGGGCAGTGATTACAGACGAGTACAAGGTGCCCAGCAAGAAGTTCAAGGTGCTGGGCAACACAGACCGCCACAGCATTAAGAAGAACCTGATTGGCGCACTGCTGTTCGACAGCGGCGAGACAGCAGAGGCAACACGCCTGAAGCGCACAGCACGCCGCCGCTACACACGCCGCAAGAACCGCATTTGCTACCTGCAGGAGATTTTCAGCAACGAGATGGCAAAGGTGGACGACAGCTTCTTCCACCGCCTGGAGGAGAGCTTCCTGGTGGAGGAGGACAAGAAGCACGAGCGCCACCCCATTTTCGGCAACATTGTGGACGAGGTGGCATACCACGAGAAGTACCCCACAATTTACCACCTGCGCAAGAAGCTGGTGGACAGCACAGACAAGGCAGACCTGCGCCTGATTTACCTGGCACTGGCACACATGATTAAGTTCCGCGGCCACTTCCTGATTGAGGGCGACCTGAACCCCGACAACAGCGACGTGGACAAGCTGTTCATTCAGCTGGTGCAGACATACAACCAGCTGTTCGAGGAGAACCCCATTAACGCAAGCGGCGTGGACGCAAAGGCAATTCTGAGCGCACGCCTGAGCAAGAGCCGCCGCCTGGAGAACCTGATTGCACAGCTGCCCGGCGAGAAGAAGAACGGCCTGTTCGGCAACCTGATTGCACTGAGCCTGGGCCTGACACCCAACTTCAAGAGCAACTTCGACCTGGCAGAGGACGCAAAGCTGCAGCTGAGCAAGGACACATACGACGACGACCTGGACAACCTGCTGGCACAGATTGGCGACCAGTACGCAGACCTGTTCCTGGCAGCAAAGAACCTGAGCGACGCAATTCTGCTGAGCGACATTCTGCGCGTGAACACAGAGATTACAAAGGCACCCCTGAGCGCAAGCATGATTAAGCGCTACGACGAGCACCACCAGGACCTGACACTGCTGAAGGCACTGGTGCGCCAGCAGCTGCCCGAGAAGTACAAGGAGATTTTCTTCGACCAGAGCAAGAACGGCTACGCAGGCTACATTGACGGCGGCGCAAGCCAGGAGGAGTTCTACAAGTTCATTAAGCCCATTCTGGAGAAGATGGACGGCACAGAGGAGCTGCTGGTGAAGCTGAACCGCGAGGACCTGCTGCGCAAGCAGCGCACATTCGACAACGGCAGCATTCCCCACCAGATTCACCTGGGCGAGCTGCACGCAATTCTGCGCCGCCAGGAGGACTTCTACCCCTTCCTGAAGGACAACCGCGAGAAGATTGAGAAGATTCTGACATTCCGCATTCCCTACTACGTGGGCCCCCTGGCACGCGGCAACAGCCGCTTCGCATGGATGACACGCAAGAGCGAGGAGACAATTACACCCTGGAACTTCGAGGAGGTGGTGGACAAGGGCGCAAGCGCACAGAGCTTCATTGAGCGCATGACAAACTTCGACAAGAACCTGCCCAACGAGAAGGTGCTGCCCAAGCACAGCCTGCTGTACGAGTACTTCACAGTGTACAACGAGCTGACAAAGGTGAAGTACGTGACAGAGGGCATGCGCAAGCCCGCATTCCTGAGCGGCGAGCAGAAGAAGGCAATTGTGGACCTGCTGTTCAAGACAAACCGCAAGGTGACAGTGAAGCAGCTGAAGGAGGACTACTTCAAGAAGATTGAGTGCTTCGACAGCGTGGAGATTAGCGGCGTGGAGGACCGCTTCAACGCAAGCCTGGGCACATACCACGACCTGCTGAAGATTATTAAGGACAAGGACTTCCTGGACAACGAGGAGAACGAGGACATTCTGGAGGACATTGTGCTGACACTGACACTGTTCGAGGACCGCGAGATGATTGAGGAGCGCCTGAAGACATACGCACACCTGTTCGACGACAAGGTGATGAAGCAGCTGAAGCGCCGCCGCTACACAGGCTGGGGCCGCCTGAGCCGCAAGCTGATTAACGGCATTCGCGACAAGCAGAGCGGCAAGACAATTCTGGACTTCCTGAAGAGCGACGGCTTCGCAAACCGCAACTTCATGCAGCTGATTCACGACGACAGCCTGACATTCAAGGAGGACATTCAGAAGGCACAGGTGAGCGGCCAGGGCGACAGCCTGCACGAGCACATTGCAAACCTGGCAGGCAGCCCCGCAATTAAGAAGGGCATTCTGCAGACAGTGAAGGTGGTGGACGAGCTGGTGAAGGTGATGGGCCGCCACAAGCCCGAGAACATTGTGATTGAGATGGCACGCGAGAACCAGACAACACAGAAGGGCCAGAAGAACAGCCGCGAGCGCATGAAGCGCATTGAGGAGGGCATTAAGGAGCTGGGCAGCCAGATTCTGAAGGAGCACCCCGTGGAGAACACACAGCTGCAGAACGAGAAGCTGTACCTGTACTACCTGCAGAACGGCCGCGACATGTACGTGGACCAGGAGCTGGACATTAACCGCCTGAGCGACTACGACGTGGACCACATTGTGCCCCAGAGCTTCCTGAAGGACGACAGCATTGACAACAAGGTGCTGACACGCAGCGACAAGAACCGCGGCAAGAGCGACAACGTGCCCAGCGAGGAGGTGGTGAAGAAGATGAAGAACTACTGGCGCCAGCTGCTGAACGCAAAGCTGATTACACAGCGCAAGTTCGACAACCTGACAAAGGCAGAGCGCGGCGGCCTGAGCGAGCTGGACAAGGCAGGCTTCATTAAGCGCCAGCTGGTGGAGACACGCCAGATTACAAAGCACGTGGCACAGATTCTGGACAGCCGCATGAACACAAAGTACGACGAGAACGACAAGCTGATTCGCGAGGTGAAGGTGATTACACTGAAGAGCAAGCTGGTGAGCGACTTCCGCAAGGACTTCCAGTTCTACAAGGTGCGCGAGATTAACAACTACCACCACGCACACGACGCATACCTGAACGCAGTGGTGGGCACAGCACTGATTAAGAAGTACCCCAAGCTGGAGAGCGAGTTCGTGTACGGCGACTACAAGGTGTACGACGTGCGCAAGATGATTGCAAAGAGCGAGCAGGAGATTGGCAAGGCAACAGCAAAGTACTTCTTCTACAGCAACATTATGAACTTCTTCAAGACAGAGATTACACTGGCAAACGGCGAGATTCGCAAGCGCCCCCTGATTGAGACAAACGGCGAGACAGGCGAGATTGTGTGGGACAAGGGCCGCGACTTCGCAACAGTGCGCAAGGTGCTGAGCATGCCCCAGGTGAACATTGTGAAGAAGACAGAGGTGCAGACAGGCGGCTTCAGCAAGGAGAGCATTCTGCCCAAGCGCAACAGCGACAAGCTGATTGCACGCAAGAAGGACTGGGACCCCAAGAAGTACGGCGGCTTCGACAGCCCCACAGTGGCATACAGCGTGCTGGTGGTGGCAAAGGTGGAGAAGGGCAAGAGCAAGAAGCTGAAGAGCGTGAAGGAGCTGCTGGGCATTACAATTATGGAGCGCAGCAGCTTCGAGAAGAACCCCATTGACTTCCTGGAGGCAAAGGGCTACAAGGAGGTGAAGAAGGACCTGATTATTAAGCTGCCCAAGTACAGCCTGTTCGAGCTGGAGAACGGCCGCAAGCGCATGCTGGCAAGCGCAGGCGAGCTGCAGAAGGGCAACGAGCTGGCACTGCCCAGCAAGTACGTGAACTTCCTGTACCTGGCAAGCCACTACGAGAAGCTGAAGGGCAGCCCCGAGGACAACGAGCAGAAGCAGCTGTTCGTGGAGCAGCACAAGCACTACCTGGACGAGATTATTGAGCAGATTAGCGAGTTCAGCAAGCGCGTGATTCTGGCAGACGCAAACCTGGACAAGGTGCTGAGCGCATACAACAAGCACCGCGACAAGCCCATTCGCGAGCAGGCAGAGAACATTATTCACCTGTTCACACTGACAAACCTGGGCGCACCCGCAGCATTCAAGTACTTCGACACAACAATTGACCGCAAGCGCTACACAAGCACAAAGGAGGTGCTGGACGCAACACTGATTCACCAGAGCATTACAGGCCTGTACGAGACACGCATTGACCTGAGCCAGCTGGGCGGCGACGCATACCCCTACGACGTGCCCGACTACGCAAGCCTGGGCAGCTAG
